# Role of the Compression Ratio in Dual-Fuel Compression Ignition Combustion with Hydrogen and Methanol

**DOI:** 10.1021/acs.energyfuels.4c02741

**Published:** 2024-09-24

**Authors:** Víctor M. Domínguez, Juan J. Hernández, Ángel Ramos, José Rodríguez-Fernández

**Affiliations:** Grupo de Combustibles y Motores, Escuela Técnica Superior de Ingeniería Industrial, Universidad de Castilla La-Mancha, Av. Camilo José Cela s/n, Ciudad Real 13071, Spain

## Abstract

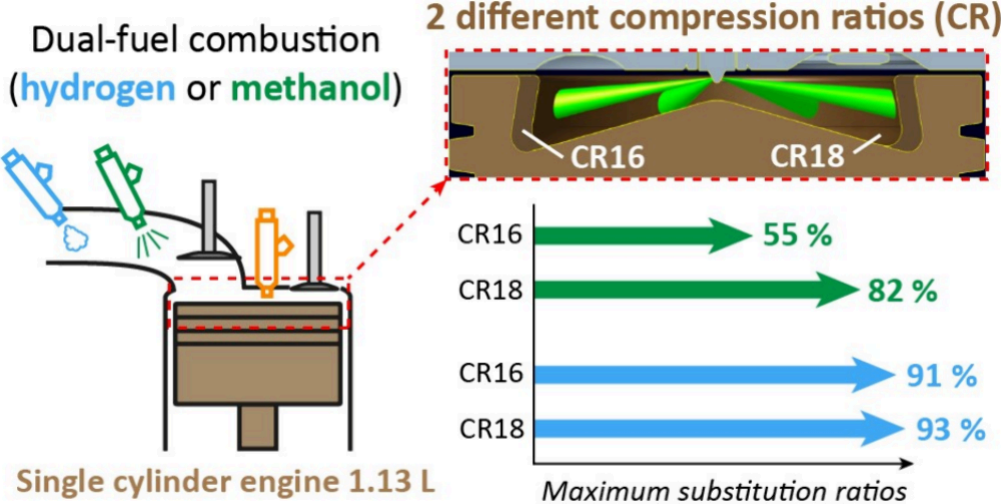

Renewable hydrogen and e-fuels, synthesized from captured CO_2_ and renewable H_2_, are feasible ways to achieve transport decarbonization, particularly in medium/heavy-duty applications and the maritime sector, where compression ignition engines predominate. Hydrogen and methanol are low carbon-intensive fuels, which can be used in these engines by carrying out dual-fuel combustion with a diesel-like fuel. Under low load engine conditions, reaching very high substitutions of the fossil diesel fuel can be a major challenge as the presence of these fuels affects negatively the autoignition process. Therefore, this work explores the substitution limits in dual-fuel mode with hydrogen and methanol under low load conditions (5.2 bar IMEP) for two different compression ratios (15.84:1 and 18.04:1) using a 1.13 L single-cylinder engine. Increasing the compression ratio allowed improvement of the maximum diesel substitution from 55 to 82% (also achieving a significant improvement of the thermal efficiency) with methanol and from 91 to 93% with H_2_ (but decreasing the thermal efficiency due to higher heat transfer losses).

## 1 Introduction

Since the late years of the past century, reducing pollutant emissions in compression ignition (CI) engines has become urgent, leading to more stringent emission standards and test procedures. More recently, achieving higher thermal efficiencies in CI engines is gaining interest following new and increasingly determined policies aimed to reduce greenhouse gas emissions.^1^

To this end, the use of sustainable, carbon-neutral (or close) fuels, coupled with more efficient combustion modes in CI engines, is the most realistic option in many sectors that are hard to decarbonize.^2,3^ Dual-fuel combustion in CI engines allows the introduction of a wide range of sustainable fuels^4^ while lowering main pollutant emissions, sometimes making unnecessary the use of aftertreatment devices^5^ that constitute a burden in terms of cost, complexity, and long-time reliability.^6^ For example, near-zero levels of soot have been reported for a wide range of equivalence ratios in dual-fuel combustion with hydrogen and diesel fuel.^7^ But, at low engine load, dual-fuel CI combustion often results in poorer efficiency compared to conventional combustion^8^ and admits very limited fossil fuel substitution ratios.^9^ Even at high loads, the maximum substitution ratio must be carefully selected to avoid the undesired preignition of the substitution fuel.

Therefore, exploring paths to improve low load operation of dual-fuel engines presents evident potential benefits. Intake air temperature and pressure,^10^ injection parameters^11^ including single/split strategies, timing or pressure, EGR rate,^12^ etc., have been explored separately, generally concluding that high EGR rates and intake charge temperature are necessary to improve the performance under this condition. Approaches searching for an integral optimization of those parameters can be found as well.^13^ Moreover, adapting the engine parameters to dual-fuel conditions may lead to an extension of the maximum substitution ratio, as Wang et al.^14^ proved by optimizing the EGR rate and combustion timing in a dual-fuel engine operated with methanol and diesel at high load.

The choice of the fuels, both the low^15^ and high^16^ reactivity fuels, also affects the efficiency and the maximum substitution ratio achievable. In a previous work,^17^ we showed that the combined use of a high-cetane number fuel such as HVO with short carbon chain alcohols led to higher substitution ratios and greater efficiency than fossil diesel. Chemical properties of the secondary fuel and its injection timing also play a role. Clean burning of hydrogen, wide flammability limits, and its emerging production route through renewable electricity make it attractive for direct use in dual-fuel CI engines,^18^ but liquid alcohols (methanol considered as an e-fuel^19^) present more energy density and ease of storage and transportation.^20^

Adjusting the effective compression ratio (CR) depending on the engine load or other operation parameters is another technique that can be used to improve the performance^21^ of CI engines operated conventionally and in dual-fuel modes. From a practical perspective, adjusting the CR is feasible through recent developments that have already come to the market,^22^ such as variable compression ratio (VCR) and variable valve timing (VVT). VCR modifies the volume of the combustion chamber as the engine is in operation,^23^ while VVT adjusts the intake valve closing timing.^24^ Applied to a reactivity-controlled compression ignition engine, Xu et al.^25^ reported that both VCR and VVT can successfully control the combustion phasing and the maximum pressure, although at high load, they found higher fuel efficiency and lower soot emission for VCR.

In dual-fuel CI engines, the effects of varying the CR go beyond the mere impact on the efficiency. Using methanol in a simulation study, Wang et al.^26^ highlighted that the equivalent ratio and temperature distributions change with the CR, which also affects the charge density, the penetration of the diesel-like fuel, and ultimately, the pollutant emission. High CR may expand the maximum limit of secondary fuel admitted and reduce particle number and mass,^27^ whereas low CR softens the pressure gradient at high load and leads to a high premixed ratio by augmenting the mixing time of the diesel-like fuel. Moreover, the higher combustion temperature associated with higher CR promotes NO*_x_* formation in conventional diesel combustion, but under lean, highly premixed combustion in dual-fuel CI engines, NO*_x_* emission is generally very low. Aydin^28^ did report higher NO*_x_* emission at higher CRs at substitution ratios below 15% using natural gas, but this was probably because for such low replacements, the combustion process did not differ much from the conventional, mostly diffusive burning of diesel fuels in CI engines.

Despite this interest, most studies on the effect of the CR in dual-fuel CI engines use simulation techniques,^26,29^ whereas experimental studies are scarce and sometimes reporting inconclusive trends. In addition, previous works focus mainly on the effect of varying CR on performance and emissions at equal or comparable substitution ratios^28^ without assessing how much the ratio of the secondary fuel can be increased by modifying the CR. In this experimental study, we evaluate this issue at low load with two promising sustainable fuels: hydrogen and methanol. These fuels have been chosen because of their increasing interest^30^ and because they were previously proved to behave differently.^9^ Hydrogen resulted in poorer performance but was able to substitute around 90% of the diesel fuel at low load, while methanol beat hydrogen in terms of efficiency, but the maximum substitution ratio was only 55%. Hence, this work contributes to broadening the soundness of the conclusions achieved.

## 2 Experimental Setup and Methodology

The experimental setup is based on a 1.13 L single-cylinder research engine, representative for medium-duty trucks, buses, and nonroad machinery, in which dual-fuel combustion with gases and liquids (as port-fuel injection fuels) can be studied. Injectors from the original engine manufacturer are used to inject port fuels: Bosch 0 280 158 862 for gases (hydrogen, in this study) and Bosch 0 280 158 040 for liquids (methanol). The engine is coupled to a Piller GMCPL 225.26B dynamometer. The installation had three independent fuel supply systems. Diesel and methanol mass flow rates were measured with two AVL 733S gravimetric balances. Hydrogen consumption was measured by using a Bronkhorst thermal mass flow meter. Injection events of these fuels were controlled by an electronic control unit (ECU), which was managed with INCA software and ETAS 592.1 hardware. Main characteristics of the engine and fuel supply systems can be found in Table 1.

**Table 1:** Technical Characteristics of the Single-Cylinder Engine and Fuel Injection Systems

cylinder bore × piston stroke	106.5 × 127 mm
cylinder volume	1131 cm^3^
maximum speed	2500 rpm
EGR system	high-pressure, cooled EGR system
valves	4 (2 inlet, 2 exhaust)
diesel injection system	direct injection, common rail (up to 2500 bar)
methanol injection system	port-fuel 4-nozzle injector, at 20 °C and 5 bar (flow rate: 187 g/min *n*-heptane at 3 bar)
hydrogen injection system	port-fuel injector, at 5 bar

**Figure 1:**
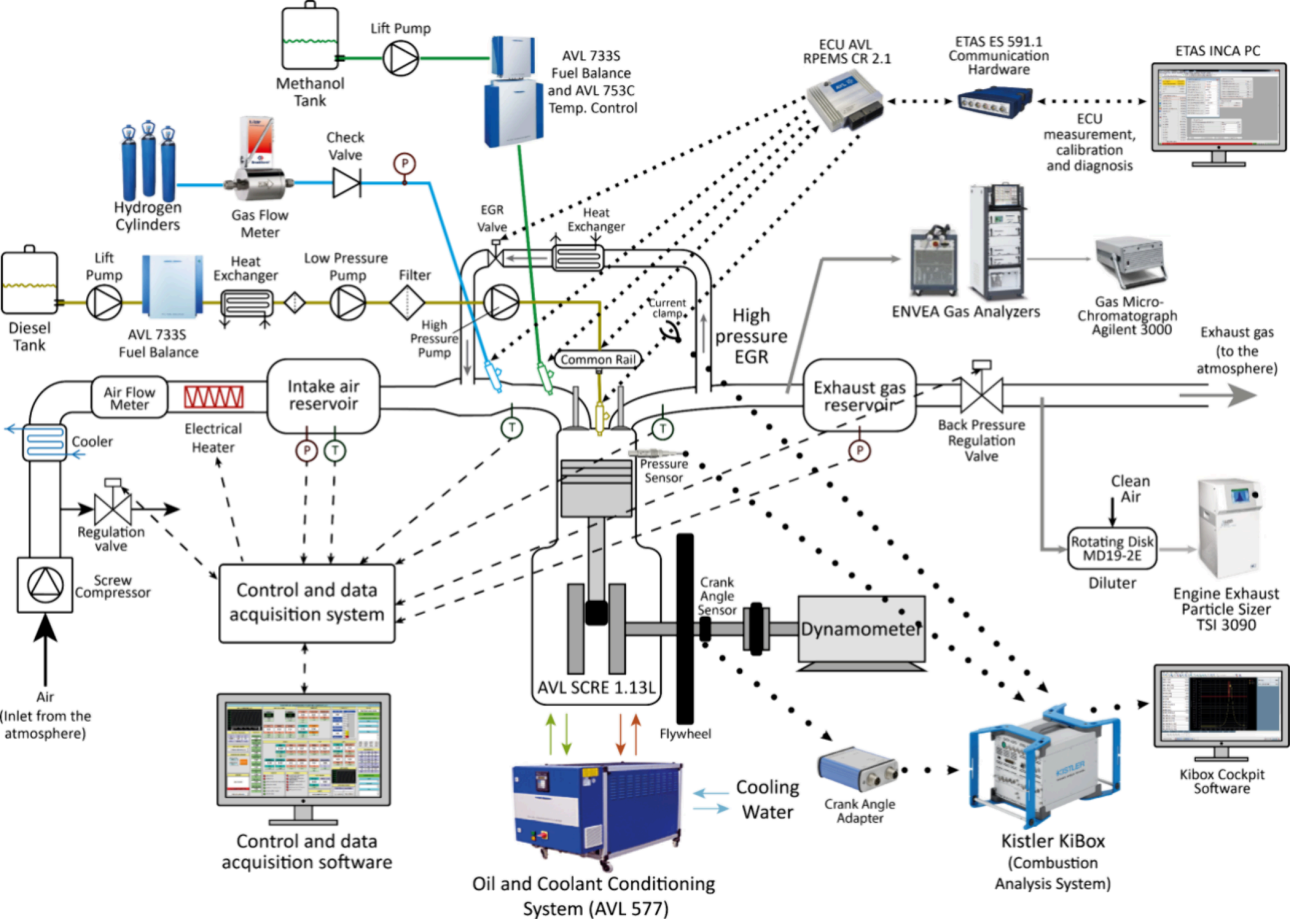
Experimental setup.

Engine operation parameters were controlled and registered through devices widely described in ref (9). A rotary piston flowmeter (Elster RVG G100) was used to measure the intake air flow rate. Figure 1 shows the scheme of the experimental setup including all control and measurement systems.

Pressure values were measured by means of PMA Gmbh P40 pressure transmitters (0–6 bar absolute pressure range). Intake air and exhaust gas reservoirs were installed (Figure 1) to avoid natural pulsation of the single cylinder engine. Temperatures were measured by fine diameter Type K thermocouples. For analyzing the combustion process and determining combustion parameters, Kibox from Kistler was employed. Based on eq 1, this system computes the apparent rate of heat release (RoHR) by capturing the in-cylinder pressure and volume using a piezoelectric pressure sensor and a crank angle encoder, respectively. Simultaneously, the energization current of the diesel injector was registered by using a current clamp to determine the injection timing.
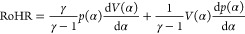
1

Gaseous emissions from the engine were measured: total hydrocarbons (THCs) through a flame ionization detector (FID), nitrogen oxides (NO*_x_*) through chemiluminescence (CLD), and carbon monoxide and carbon dioxide (CO and CO_2_) with a nondispersive infrared detector (NDIR). Additionally, a microgas chromatograph (Agilent 3000 series) was employed to measure unburnt hydrogen in the exhaust. Gas analyzer measurement accuracy is 1% of full scale. As described previously,^9^ an engine exhaust particle sizer (EEPS) spectrometer, model 3090 from TSI, and a two-stage dilution system with a total dilution factor of 227:1 were used to measure particle size distributions.

The diesel fuel used was donated by Repsol. It is a first-fill diesel fuel with no oxygenates or any other renewable fraction. Hydrogen cylinders (purity higher than 99.999%) were acquired from Air Liquide, while methanol (with a purity above 99.5%) was provided by Panreac. Main properties of these fuels are listed in Table 2.

**Table 2:** Fuel Properties

	diesel	methanol	hydrogen
density at 15 °C (kg/m^3^)	826.6	796.1	0.089^a^
lower heating value (MJ/kg)	42.9	20.1	120.8
derived cetane number	59.85	3	
C/H/O (% w/w)	86.4/13.6/0	37.5/12.5/50	0/100/0
molecular weight (g/mol)	203	32.04	2.02
stoichiometric air-to-fuel ratio	14.56	6.46	34.46

a Under normal conditions.

**Figure 2:**
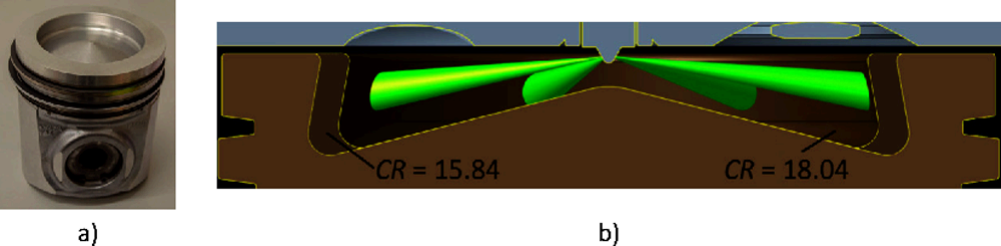
Piston picture (a) and design for each compression ratio (b).

To extend the substitution ratio range, the compression ratio of the engine was increased. For this purpose, the base piston (centered toroidal bowl in piston, see Figure 2a), with a compression ratio of 15.84:1, was replaced by a similar one with a smaller bowl volume (Figure 2b) to get a CR of 18.04:1. From now on, compression ratios of 18.04:1 and 15.84:1 are denoted as CR18 and CR16, respectively. Combustion chamber volumes at the TDC were 76.2 (CR16) and 66.4 cm^3^ (CR18). All tests were performed at low load. The selected steady-state mode was defined by an IMEP of 5.2 bar and an engine speed of 1600 rpm. These conditions were selected to replicate 25% of full load at intermediate speed based on the 13-mode test for medium- and heavy-duty trucks considered by UNECE global technical regulation no. 4.^31^

The substitution ratio (SR), in terms of energy, is calculated through eq 2, where the mass flow rates (*ṁ*) and lower heating values (LHV) of the two fuels are used. PFI and DI refer to the port-injection fuels (hydrogen or methanol) and direct-injection fuels (diesel fuel), respectively. For coding, MXD and HXD refer to substitution with an *X* percentage of methanol or hydrogen, respectively.
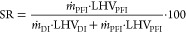
2

Energy balances were performed based on eq 3, where the fuel energy flow rate (*Ė*_f_) was obtained from eq 4 and the indicated power (*N*_*i*_) through eq 5, which uses the swept volume (*V*_D_), the engine speed (*n*), and the IMEP value. Exhaust enthalpy flow rate (*Ḣ*_exh_) was calculated with eq 6 using the exhaust mass flow rate (*ṁ*_exh_), the specific heat capacity of air (*c*_p,air_, which is assumed to be equal to that of the exhaust gas), and the difference between the exhaust and intake temperatures (*T*_exh_ and *T*_int_, respectively). The power loss associated with the unburnt fuel (*Ḣ*_unburnt_) was calculated through the mass flow rates of H_2_, CO, and THC, measured in the exhaust gas through the analyzers above explained, and their respective lower heating values using an equation similar to eq 4. For dual-fuel tests with methanol, all THCs were assumed to be unburnt methanol. In dual-fuel mode with H_2_ and conventional mode tests, THCs were assumed to be unburned diesel fuel. Heat transfer loss (*Q̇*_HT_) was deduced from eq 3. Finally, the engine-indicated thermal efficiency (ITE) can be calculated according to eq 7 using the indicated power and the fuel energy flow rate (*Ė*_f_).
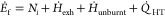
3
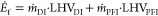
4
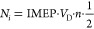
5
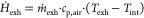
6
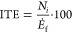
7

Regarding combustion parameters, the combustion duration was calculated as the angle interval between α_5_ and the end of combustion indicated by α_80_ (as in another work^32^), with α_80_ and α_5_ being the crank angles at which 80 and 5% of total apparent heat have been released, respectively. The ignition delay was defined as the crank angle between the start of the diesel pilot injection and the value of α_5_. The coefficient of variation (COV) of IMEP was calculated as the ratio of the standard deviation to the mean using 100 consecutive thermodynamic cycles. It should be noted that some restrictions were considered to ensure safe operation and engine integrity, such as maximum limits for COV of IMEP (4%), in-cylinder pressure (190 bar), and pressure gradient (15 bar/°CA). All experiments carried out in this work were replicated twice to guarantee repeatability.

For both compression ratios, the substitution ratio was increased in steps of 5–20% (in general, beginning with larger steps in the low SR region and moving to smaller steps when getting close to the maximum substitution attainable with each PFI fuel) until the substitution limits were reached. The engine operating conditions used in the experiments were fixed (intake pressure and temperature of 1.3 bar and 35 °C, respectively, dwell angle of 10 °CA, exhaust backpressure of 1.45 bar, and diesel injection strategy split into pilot and main injections) according to previous works.^9,33^ In those works, two-stage optimization processes (first, a fractional factorial design and then a response surface methodology) were carried out for diesel-methanol and diesel-hydrogen dual-fuel combustion with a compression ratio of CR16. For CR18, the same optimized operating conditions as those for CR16 were replicated.

The main conclusion of these optimization processes was that the dual-fuel mode required more centered combustion with higher EGR rates and lower injection pressures than conventional diesel combustion. Thus, only the exhaust gas recirculation (EGR) rate, the combustion phasing (α_50_, crank angle at which 50% of total apparent heat has been released, which can be controlled by modifying the diesel injection timing), and the diesel injection pressure (*p*_inj_) were different among the tests here carried out, as it will be shown in the results.

## 3 Results and Discussion

### 3.1 Substitution Limits

**Figure 3:**
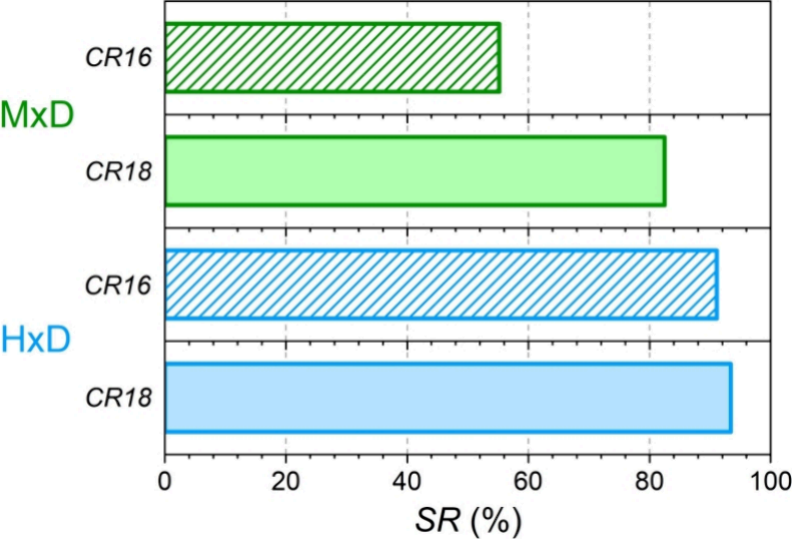
Maximum energy substitution ratios.

**Figure 4:**
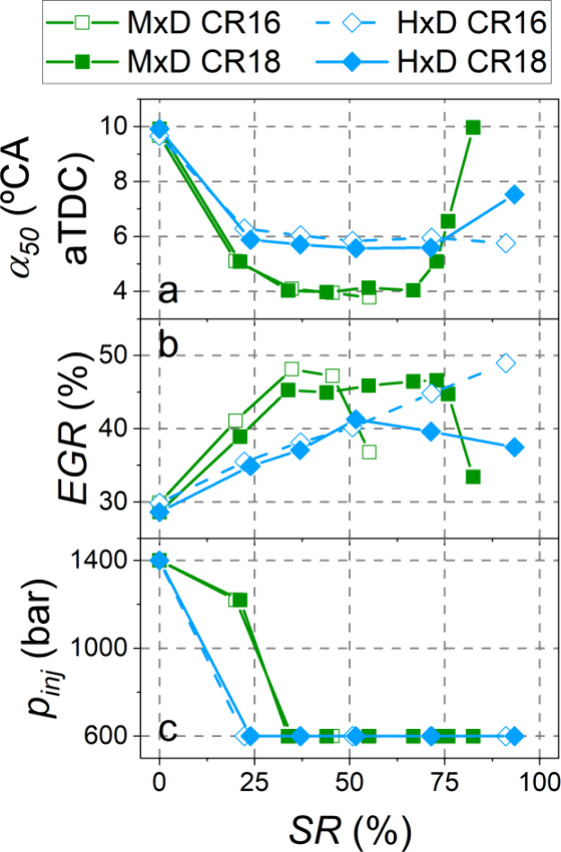
α_50_ (a), EGR rate (b), and injection pressure (c).

Figure 3 summarizes the maximum substitution achieved for each fuel combination and compression ratio. Figure 4 shows the combustion phasing, EGR rate, and diesel injection pressure in each test, which were different as the SR was changed according to the findings of previous work^9^ aimed to improve the performance and emissions of the engine operated at CR16 under conventional and dual-fuel combustion. Under dual-fuel mode and up to 45% substitution ratio (methanol) and 50% (hydrogen), the values of these parameters were very similar for CR18 and CR16. Clearly, dual-fuel combustion required larger EGR rates, more centered combustion, and lower injection pressure than conventional, diesel-only combustion.

Methanol in dual-fuel mode significantly increased the diesel ignition delay (as described in the following section) due to its cooling effect and low cetane number. When high methanol substitution ratios were tested, the ignition delays were so long that diesel autoignition failed. As a result, with CR16, it was possible to achieve only 55% SR with methanol and at the expense of a lower EGR rate (Figure 4 b) to ensure diesel autoignition. With CR18, it was possible to increase the maximum methanol substitution ratio up to 82%, significantly higher than with CR16. This is justified because the more favorable in-cylinder conditions (pressure and temperature) at the end of the compression stroke lessened the cooling effect of methanol evaporation and favored diesel autoignition. With CR18 and methanol, optimal operating conditions (EGR rate around 45%, combustion center at 4 °CA aTDC) were maintained in the medium substitution range up to SR = 70%. From this ratio to the maximum shown in Figure 3 (82%), searching for maximizing the diesel replacement, combustion had to be delayed and the EGR rate decreased to limit the ignition delay, thus preserving diesel autoignition and a stable operation.

In the case of hydrogen dual-fuel mode tests, the operating conditions were similar for both compression ratios up to SR = 50% (Figure 4). For higher substitutions and with CR18, hydrogen burns faster, leading to a more intense combustion with higher pressure gradients (as will be shown in the following section), which compromise the engine safety. Therefore, it was necessary to decrease the EGR rate (this reduced the premixed combustion fraction) and to slightly delay α_50_. When using hydrogen in dual-fuel combustion mode, increasing the compression ratio increases the maximum substitution ratio by only 2%, as hydrogen hardly impedes the diesel autoignition. At both compression ratios, the energy substitution ratio with hydrogen exceeds 90%, clearly superior to the values achieved with methanol.

### 3.2 Combustion Diagnosis

**Figure 5:**
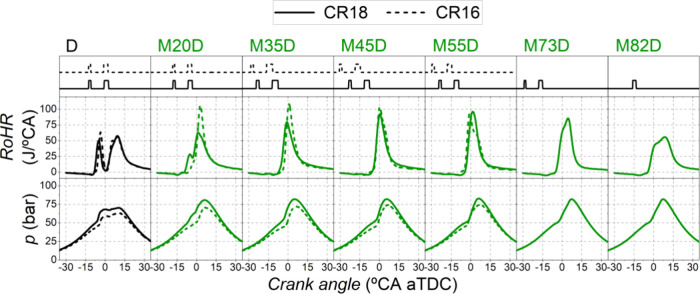
RoHR and in-cylinder pressure. Dual-fuel mode with methanol at both compression ratios.

**Figure 6:**
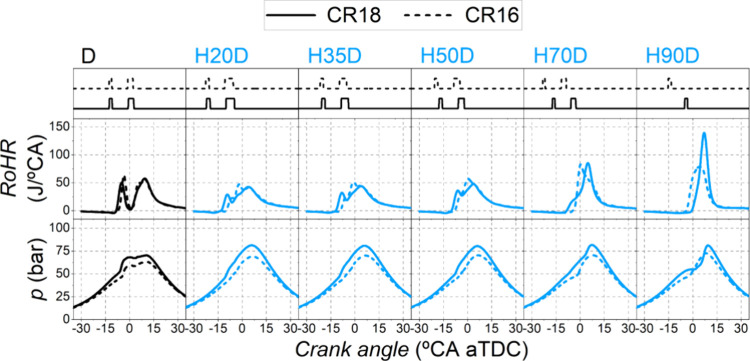
RoHR and in-cylinder pressure. Dual-fuel mode with hydrogen at both compression ratios.

Figures 5 and 6 show the in-cylinder pressure and the rate of heat release. As expected, higher maximum cylinder pressures were achieved with the highest compression ratio (CR18). Also, by increasing the compression ratio from 15.84:1 to 18.04:1, the pressure and temperature conditions in the combustion chamber were more favorable both for the diesel-fuel to achieve autoignition and for the PFI fuel to promote more robust turbulent flame propagation.

**Figure 7:**
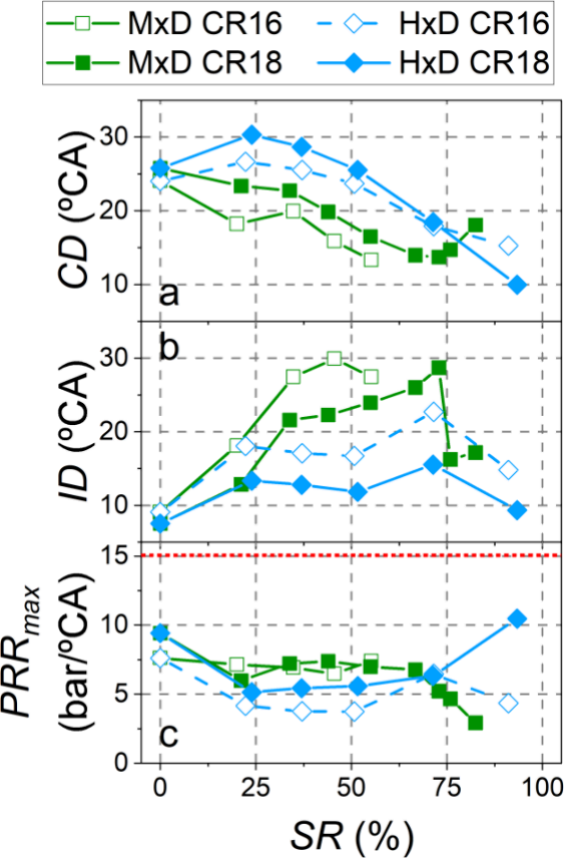
Combustion diagnosis parameters (the red dotted line marks the maximum permissible limit).

As mentioned in the previous section, methanol hindered diesel-fuel autoignition by increasing the ignition delay due to the low reactivity of methanol and its cooling effect. The lower compression ratio (CR16) makes it even more difficult to achieve diesel autoignition when methanol was used. Therefore, with CR16, the ignition delays were longer than with CR18 (Figure 7b) and, consequently, higher premixed ratios were achieved, leading to a shorter combustion duration (Figure 7a) and to higher peaks in the rate of heat release. In fact, for SR = 20%, it is observed that with CR18, the diesel pilot injection autoignites separately from the main injection, unlike with CR16. In the experiments with substitutions above 60% (methanol and CR18), flame propagation in the combustion chamber became dominant, which caused the CD to increase. It should be noted that with SR > 75%, the pilot injection could not be maintained (the injector could not be opened in such short periods) and, therefore, the ignition delays were shorter for these experiments.

The main difference between the two compression ratios when hydrogen was used at low substitution ratios was the combustion of the diesel pilot injection. With CR18, the preinjected diesel fuel burned much earlier than the start of the main injection, resulting in a shorter ignition delay (Figure 7b), longer combustion duration (Figure 7a), and a higher fraction of the total heat release before TDC. At high substitution ratios (SR > 70%), as with methanol, the rate of heat release pattern changed to modes governed by hydrogen flame propagation through the combustion chamber. The higher temperatures and pressures when using CR18 enhanced such flame propagation inside the combustion chamber (increasing also the hydrogen flame speed^34^). This caused a very rapid combustion at maximum substitution (SR = 93%) with a very high RoHR peak. Furthermore, the EGR rate had to be reduced (Figure 4b) and combustion delayed (Figure 4a) with the aim of not surpassing the PRR_max_ limit (15 bar/°CA) set to preserve the engine durability. Even with these adjustments, the maximum pressure rise rate was significant, at around 10 bar/°CA, as shown in Figure 7c.

**Figure 8:**
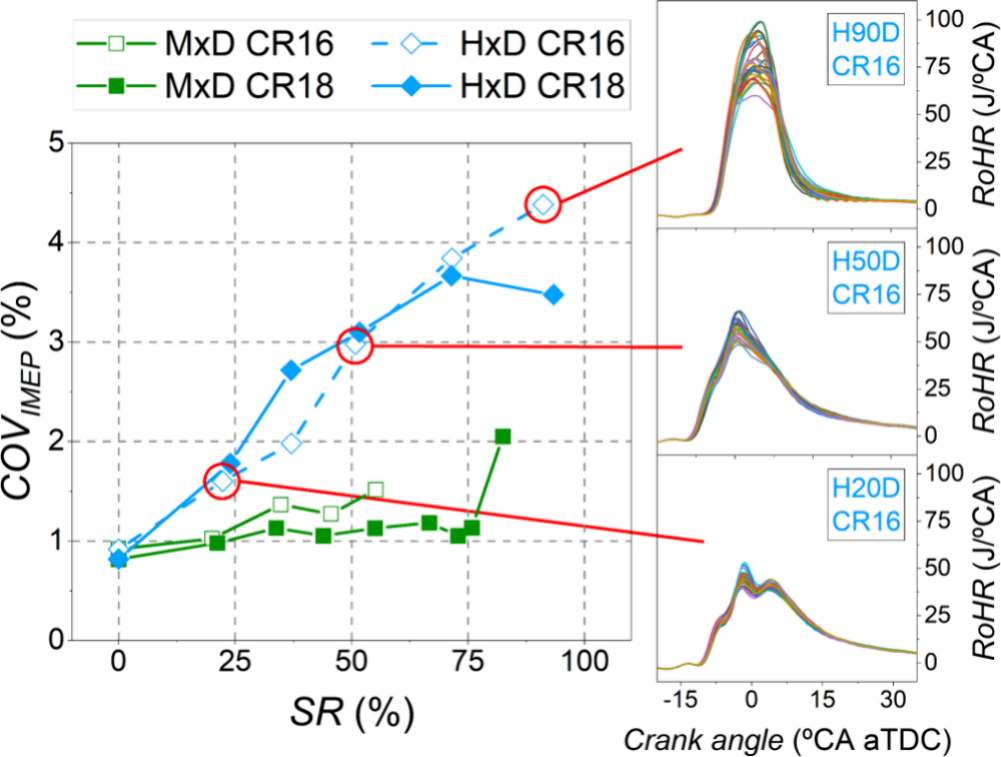
Coefficient of variation (COV) of IMEP and examples of cyclic dispersion in RoHR curves (30 consecutive thermodynamic cycles for H20D, H50D, and H90D with CR16).

Combustion stability was evaluated by the coefficient of variation of the indicated mean effective pressure (COV_IMEP_), shown in Figure 8 along with 30 consecutive RoHR curves recorded in KIBOX for SR values of 20, 50, and 90% (H20D, H50D, and H90D; only for hydrogen as it was the PFI leading to the most unstable combustion, as it is described below). While the compression ratio had no significant effect on COV_IMEP_, a clear difference was observed between the two PFI fuels. When methanol was used, there was little difference in engine stability compared to the conventional mode, although slightly higher COV_IMEP_ values were observed for methanol modes. However, in dual-fuel combustion with H_2_, increasing the energy substitution ratio made the engine run more unstably, as also reported by other authors at low load.^35,36^ As Sharma and Dhar^35^ pointed out, the combined use of two fuels with different ignition properties, as it occurs in dual-fuel CI combustion, tends to increase the cycle-to-cycle variability. For lean combustion, it is also suggested that the combustion instability is higher with nonhomogeneous mixtures. Since the ignition delay is shorter with hydrogen compared to methanol (Figure 7b), this would support the higher COV_IMEP_ observed with hydrogen. Additionally, Liew et al.^37^ reported that at low load, some unburnt hydrogen remains inside the cylinder as part of the residual mass. This hydrogen mass can intermittently participate in combustion due to its higher flammability, resulting in increased combustion variability. For the maximum substitution ratios with H_2_, COV_IMEP_ values close to 4% were achieved, which was considered in this work as the limit value to ensure the stable operation of the engine.

### 3.3 Engine Performance

**Figure 9:**
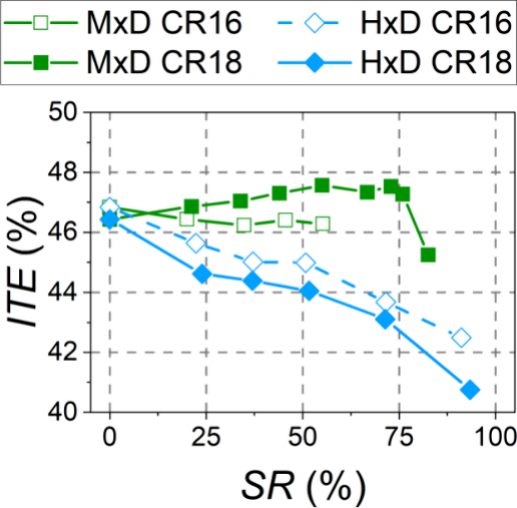
Effect of the substitution ratio on engine-indicated thermal efficiency at both compression ratios.

**Figure 10:**
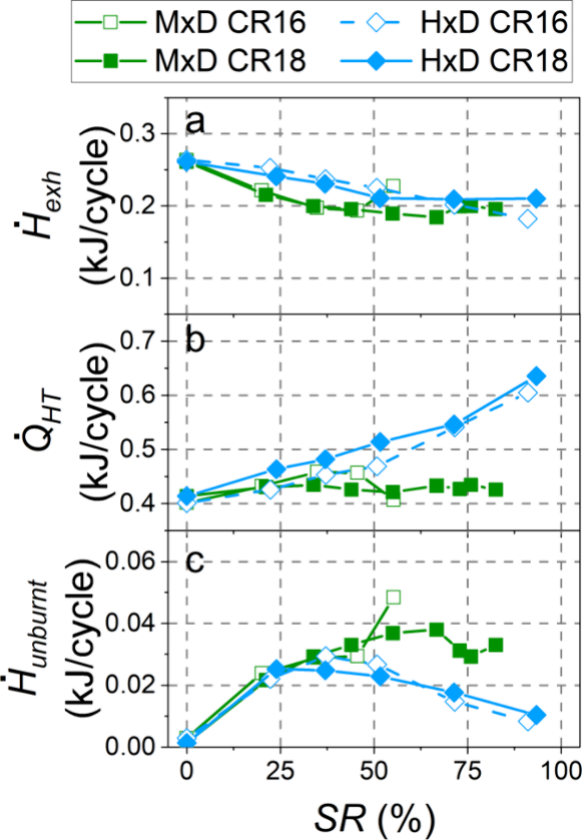
Exhaust gas enthalpy flow (a), power loss from heat transfer (b), and power loss from the unburnt fuel (c).

The effect of the compression ratio and substitution ratio with methanol and hydrogen on the indicated thermal efficiency (ITE) is shown in Figure 9. Figure 10 breaks down the energy balance components: the exhaust enthalpy flow rate (*Ḣ*_exh_), heat transfer losses (*Q̇*_HT_), and the power loss from the unburnt fuel (*Ḣ*_unburnt_). Since the IMEP (5.2 bar) and engine speed (1600 rpm) were fixed in all experiments, the indicated power was always the same (7.6 kW), and it is not included in Figure 10.

Trends in ITE under dual-fuel combustion with methanol were opposite of the two compression ratios. With CR16, ITE slightly decreased compared to that in the conventional combustion mode. In contrast, when CR18 was used, ITE increased more than one percentage point compared to the conventional mode for a wide range of substitution ratios (35–75%). When this alcohol is used, higher unburned losses and lower exhaust losses (the latter due to the higher EGR rates used) were revealed compared to those in the conventional mode, regardless of the compression ratio. The main difference in the energy balance between CR18 and CR16 was found in the heat transfer losses for SR = 35% and SR = 45%. While these losses remained similar to the conventional combustion mode for CR18, they increased considerably for CR16, probably due to the higher temperature achieved in the combustion chamber due to the shorter combustion duration. Certainly, the RoHR peaks were higher for CR16 (Figure 5). It should be noted that the test with the highest substitution ratio achieved (82%, CR18) had a low ITE because the combustion was significantly delayed, as the large amount of alcohol hindered advancing the combustion process.

For hydrogen, ITE markedly decreased with an increase in the energy substitution ratio for both CR18 and CR16. By increasing the SR, the exhaust enthalpy flow rate decreased (due to the lower exhaust mass flow rate as a consequence of the low density of H_2_) and the onset of turbulent flame propagation led to a better combustion efficiency (lower power loss from the unburnt fuel). However, the high hydrogen flame temperature and the faster heat release caused higher heat transfer losses through the cylinder walls. Hydrogen flames have a quenching gap approximately three times smaller than that of other fuels such as gasoline,^38^ so turbulent H_2_ flames approach closer to the cylinder walls before extinguishing. The reduction in *Ḣ*_unburnt_ and *Ḣ*_exh_ (Figure 10a,c) did not outweigh the increase in heat transfer losses (Figure 10b), which caused the ITE to diminish. This decrease in ITE was even more pronounced for replacements above 70%, where turbulent flame propagation throughout the combustion chamber predominated.

In regard to the compression ratio, CR18 achieved lower ITE than CR16 for the same substitution ratio, possibly because of the more significant weight of the heat released before TDC due to an earlier start of combustion. Probably, a shorter dwell angle could reduce this effect. For example, at SR = 35%, less than 3% of the total heat was released before −5 °CA aTDC with CR16, while almost 11% was released with CR18. Also, with CR18, the in-cylinder pressure and temperature inside the chamber were higher, which favored heat transfer to the walls (Figure 10 b), possibly due to the higher pressure reducing the quenching distance.^39^

### 3.4 Exhaust Emissions

**Figure 11:**
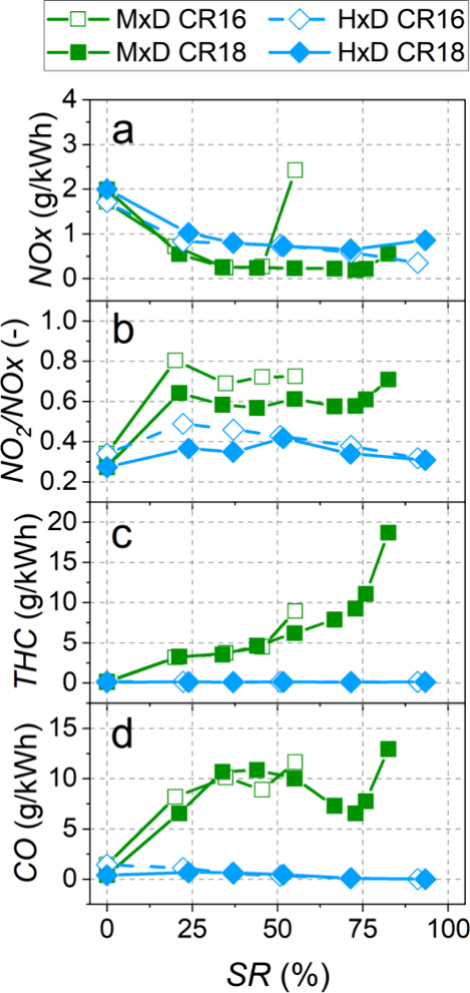
Effect of the substitution ratio on NO*_x_* (a), NO_2_/NO*_x_* ratio (b), THC (c), and CO (d).

The effects of the substitution ratio and compression ratio on gaseous emissions are shown in Figure 11. NO*_x_* emissions were lower under dual-fuel combustion with methanol due to the increased EGR rate (45–48%, compared to 30% in the conventional combustion mode) and the cooling effect caused by the methanol evaporation. By using such high EGR rates, it was possible to advance combustion (close to TDC) to increase the ITE with little penalty for NO*_x_* emission. In addition, exhaust gas recirculation allowed the reburning of unburned methanol. The exception was the highest methanol substitution ratio (55%) using CR16, which resulted in NO*_x_* emissions higher than conventional diesel combustion, due to the limited EGR rate (Figure 4b) that was necessary to sustain the combustion process. In dual-fuel combustion with H_2_, NO*_x_* emission was also lower than in conventional combustion because of the higher EGR rates (Figure 4b). Thus, the NO*_x_* emission was more influenced by the PFI fuel and the EGR rates than by the compression ratio. However, the compression ratio did affect the NO_2_/NO*_x_* ratio: in general, CR18 resulted in a lower NO_2_/NO*_x_* ratio for conventional and dual-fuel combustion because the higher compression and combustion temperatures achieved with CR18 promoted the thermal NO formation mechanism. In addition, both methanol and hydrogen dual-fuel combustion clearly increased this ratio compared to conventional combustion, more substantially in the case of methanol. This result has been associated with a higher production of HO_2_ radicals when using H-rich fuels,^40^ which subsequently react with NO, enhancing the conversion to NO_2_.^41^ This trend has been confirmed both experimentally and through CFD models.^41^

THC and CO emissions increased significantly when using methanol in dual-fuel combustion mode (Figure 11c,d) at both compression ratios. THC emissions, which were mainly unburnt methanol, were very similar for the two compression ratios. These emissions increased gradually with the presence of alcohol in the combustion chamber. This is supported by the lower combustion temperature due to the high latent heat of vaporization of methanol, which hindered the propagation of turbulent flames through the combustion chamber, resulting in a less complete combustion. As mentioned above, when methanol was used, a significant increase in CO emissions was also observed, which was due to incomplete combustion. In conventional diesel mode and dual-fuel mode with low methanol substitution ratios, lower CO emissions were achieved for CR18 due to higher in-cylinder temperatures. In contrast, when using intermediate and high substitution ratios, CO emissions were more affected by the differences in EGR rates (a bit higher with CR16, which resulted in higher CO emissions) than by the compression ratio.

**Figure 12:**
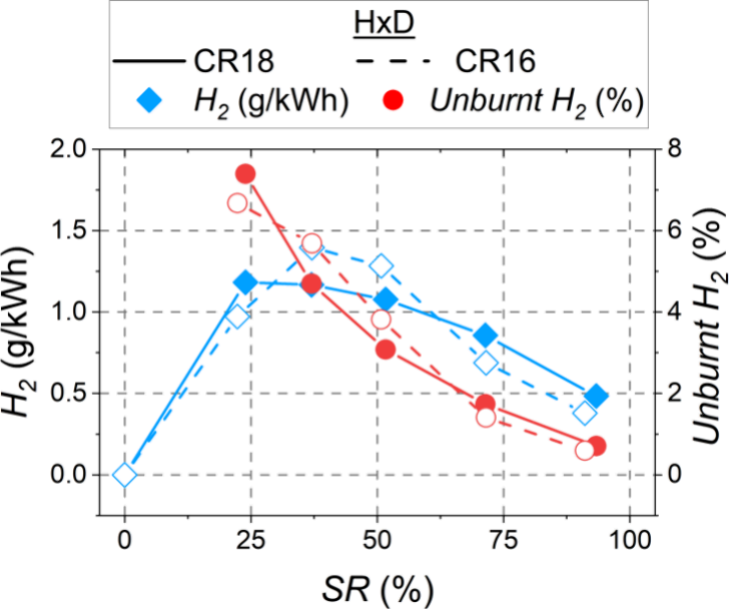
Effect of hydrogen substitution ratio on specific H_2_ emission and percentage of unburned H_2_.

When hydrogen was used in dual-fuel mode, the CO and THC emissions were almost zero, as this gaseous fuel has no carbon in its composition. Even though the dual-fuel mode with H_2_ achieved very low CO and THC emissions, it is noted in Figure 10c that power losses from the unburnt fuel were much higher than those of the conventional mode, especially at low substitution ratios. This is due to unburnt hydrogen, which is shown in Figure 12 along with the percentage of unburnt H_2_ (hydrogen in the exhaust gas with respect to that fed into the cylinder). For both compression ratios, approximately 7% of the introduced hydrogen failed to burn at SR = 20%. It could be observed that the percentage of unburnt H_2_ decreased with increasing substitution ratio for both compression ratios, reaching values below 1% for SR = 90%. This decrease in hydrogen emission was due to the higher H_2_ concentration in the combustion chamber, which allowed more regions to be within the flammability range. This facilitated the propagation of turbulent flames throughout the lean H_2_/air mixture, thus involving a larger amount of H_2_ in the combustion process.

When comparing between both compression ratios, for SR = 35–45% (same α_50_ and EGR with both CR), lower H_2_ emission was achieved for CR18, since the higher temperatures in the combustion chamber promoted flame propagation. In the experiments with 70 and 90% hydrogen, the greater H_2_ emission with CR18 was due to the decrease in the EGR rate to avoid excessive pressure rise rates.

**Figure 13:**
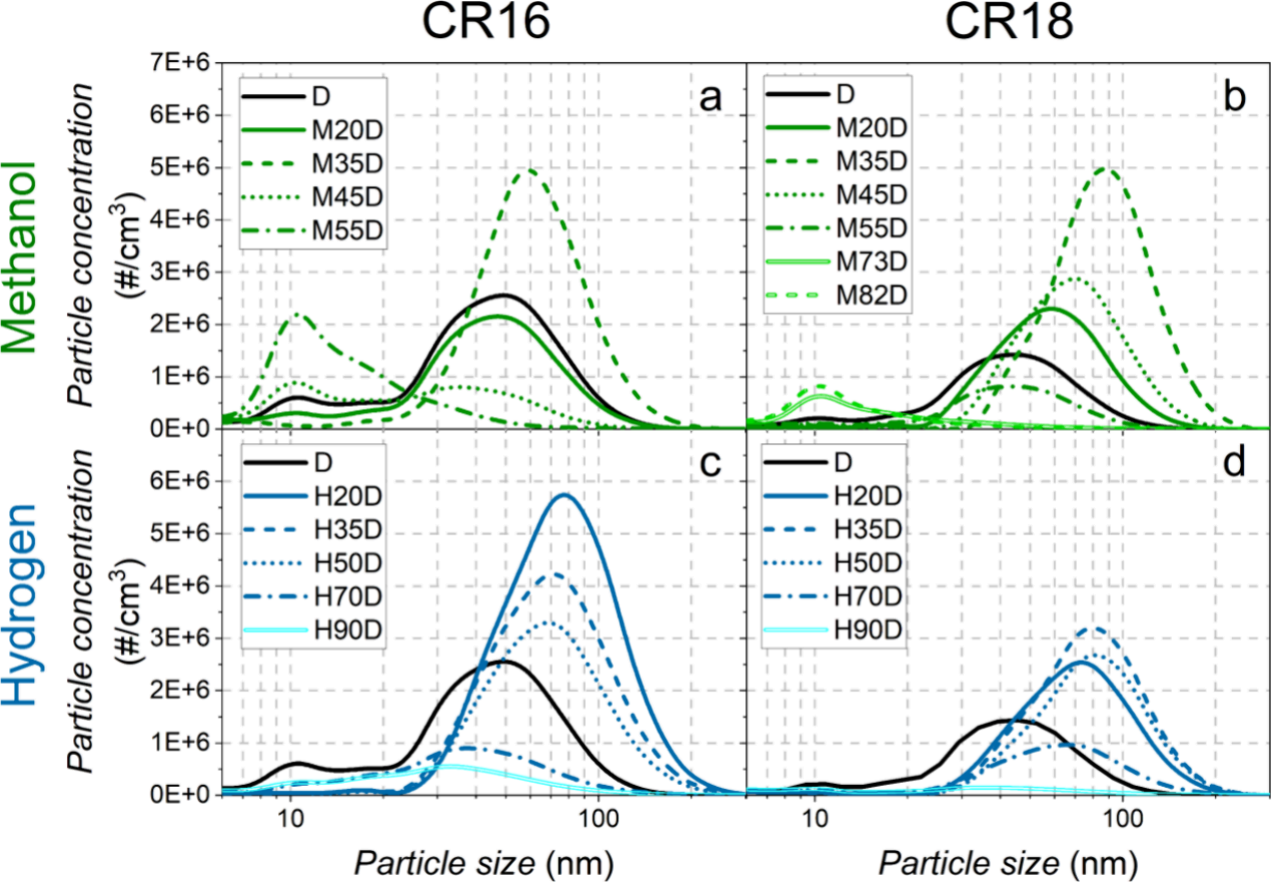
Particle size distribution.

Particle size distributions are displayed in Figure 13. For low substitution ratios (up to SR = 35%), the higher EGR rates (Figure 4b) and the lower diesel injection pressure (Figure 4c) led to more particles for all conditions. For higher substitution ratios, the results evidenced that particle emissions are a competition between soot formation and oxidation.^42^ However, the greater amount of a carbon-free fuel (hydrogen) or low-carbon fuel (methanol), together with the significant oxygen content in the case of methanol, led to an almost soot-free combustion for SR values higher than 35%. This latter trend was also supported by a more premixed combustion process when SR increases.

In regard to the effect of the compression ratio on particle emissions, the higher in-cylinder temperatures for CR18 promoted particle oxidation and consequently decreased soot emission compared to CR16. In dual-fuel mode with methanol for intermediate substitution ratios (20–45%), a higher soot emission was obtained with CR18. It could be explained by the difference in the combustion pattern (combustion processes with a lower premixing rate and shorter delay times with CR18, compared to CR16), which showed a more significant effect than the increase in the in-cylinder temperature due to the higher compression ratio. For the highest methanol substitution ratios, particle emission between 30 and 120 nm was almost zero with a peak centered in the 10–20 nm range (nucleation mode), which was higher with CR16. This emission was mainly due to the nucleation of hydrocarbons present in the exhaust gases.

## 4 Conclusions

The effect of the compression ratio in a dual-fuel engine has been evaluated, disclosing a strong dependence on the PFI fuel used, methanol or hydrogen.

By changing from CR16 to CR18, methanol allowed increasing the maximum ratio from 55 to 82%, accompanied by a significant improvement in the engine thermal efficiency. Moreover, methanol and CR18 were the only combination of PFI fuel and compression ratio that successfully increased the thermal efficiency compared to conventional (only diesel) combustion. It was concluded that the more favorable thermodynamic conditions in the combustion chamber with CR18 clearly compensated for the cooling effect caused by methanol evaporation, thus shortening the ignition delay and facilitating diesel autoignition. Both higher methanol substitution ratios and improved efficiency in dual-fuel CI engines are decisive contributions toward decreasing GHG emissions in those sectors that are heavily dependent on this type of engine. Even more, CR18 did not increase NO*_x_* emissions compared to CR16, confirming that methanol combustion at mid- and high substitution ratios reduces NO*_x_* by 80%, approximately, compared to diesel combustion and regardless of the compression ratio.

Oppositely, the use of hydrogen as PFI fuel did not provide any significant benefit. The maximum H_2_ substitution ratio, which is higher than for methanol, only increased from 91% (CR16) to 93% (CR18), but the indicated thermal efficiency decreased as a consequence of the higher heat transfer losses to the cylinder wall. In addition, with CR18, the pressure rise rates were higher, which could be a threat to engine durability. It could be interesting to reoptimize the engine operating conditions for the dual-fuel mode with hydrogen for CR18. Also, increasing the substitution ratio with H_2_ resulted in an increase in engine instability regardless of the CR used.

## References

[ref1] Islam Rony Z.; Mofijur M.; Hasan M. M.; Rasul M. G.; Jahirul M. I.; Forruque Ahmed S.; Kalam M. A.; Anjum Badruddin I.; Yunus Khan T. M.; Show P. L. Alternative fuels to reduce greenhouse gas emissions from marine transport and promote UN sustainable development goals. Fuel 2023, 338, 127220 10.1016/j.fuel.2022.127220.

[ref2] Mangesh V. L.; Tamizhdurai P.; Vedavalli R.; Santhosh S.; Kumaran R.; Siva Kumar N.; Al-Fatesh A. S.; Kumar Basivi P.; Murali G. Maritime decarbonization: Alternate marine fuel from hydroprocessing of waste plastics. Fuel 2024, 365, 131233 10.1016/j.fuel.2024.131233.

[ref3] Almena A.; Siu R.; Chong K.; Thornley P.; Röder M. Reducing the environmental impact of international aviation through sustainable aviation fuel with integrated carbon capture and storage. Energy Conversion and Management 2024, 303, 118186 10.1016/j.enconman.2024.118186.

[ref4] Hernández J. J.; Cova-Bonillo A.; Ramos Á.; Wu H.; Rodríguez-Fernández J. Autoignition of sustainable fuels under dual operation with H2-carriers in a constant volume combustion chamber. Fuel 2023, 339, 127487 10.1016/j.fuel.2023.127487.

[ref5] Benajes J.; Pastor J. V.; García A.; Boronat V. A RCCI operational limits assessment in a medium duty compression ignition engine using an adapted compression ratio. Energy Conversion and Management 2016, 126, 497–508. 10.1016/j.enconman.2016.08.023.

[ref6] Liao J.; Wang Z.; Hu J.; Yan F.; Wu Y.; Cai Z.; Zheng S.; Li S.; Peng H. Investigation of the effect of different structure parameters and operating factors on the integrated exhaust aftertreatment system for diesel engines and parameter importance analysis. Journal of Cleaner Production 2024, 447, 141257 10.1016/j.jclepro.2024.141257.

[ref7] Tomita E; Kawahara N.; Piao Z.; Fujita S Hydrogen combustion and exhaust emissions ignited with diesel oil in a dual fuel engine. SAE Technical Paper 2001, 2001–01–3503.

[ref8] Dimitriou P.; Tsujimura T.; Suzuki Y. Low-load hydrogen-diesel dual-fuel engine operation – A combustion efficiency improvement approach. Int. J. Hydrogen Energy 2019, 44, 17048–17060. 10.1016/j.ijhydene.2019.04.203.

[ref9] Domínguez V. M.; Hernández J. J.; Ramos Á.; Reyes M.; Rodríguez-Fernández J. Hydrogen or hydrogen-derived methanol for dual-fuel compression-ignition combustion: An engine perspective. Fuel 2023, 333, 126301 10.1016/j.fuel.2022.126301.

[ref10] Kumar K. S.; Raj R. T. K. Effect of Fuel Injection Timing and Elevated Intake Air Temperature on the Combustion and Emission Characteristics of Dual Fuel Operated Diesel Engine. Procedia Engineering 2013, 64, 1191–1198. 10.1016/j.proeng.2013.09.198.

[ref11] Zeng W.; Sun W.; Guo L.; Zhang H.; Yan Y.; Lin S.; Zhu G.; Jiang M.; Yu C. Optical investigation on effects of diesel injection strategy on ammonia/diesel dual fuel combustion characteristics and flame development. Fuel 2024, 363, 131027 10.1016/j.fuel.2024.131027.

[ref12] Elkelawy M.; El Shenawy E. A.; Mohamed S. A.; Elarabi M. M.; Alm-Eldin Bastawissi H. Impacts of EGR on RCCI engines management: A comprehensive review. Energy Conversion and Management 2022, 14, 100216 10.1016/j.ecmx.2022.100216.

[ref13] Yang D.; Wei S.; Ma Y.; E J.; Zhao J. Influence of critical parameters on combustion and emission characteristics of methanol/diesel dual fuel compression combustion engine. Fuel 2024, 368, 131647 10.1016/j.fuel.2024.131647.

[ref14] Wang B.; Yao C.; Chen C.; Feng J.; Lu H.; Feng L. To extend the operating range of high MSP with ultra-low emissions for DMDF unit pump engine. Fuel 2018, 218, 295–305. 10.1016/j.fuel.2018.01.028.

[ref15] Chen Z.; He J.; Chen H.; Geng L.; Zhang P. Comparative study on the combustion and emissions of dual-fuel common rail engines fueled with diesel/methanol, diesel/ethanol, and diesel/n-butanol. Fuel 2021, 304, 121360 10.1016/j.fuel.2021.121360.

[ref16] Rodríguez-Fernández J.; Ramos Á.; Domínguez V. M.; Giménez B.; Reyes M.; Hernández J. J. Hydrogen use in a dual-fuel compression-ignition engine with alternative biofuels. Johnson Matthey Technology Review 2024, 68, 381–395. 10.1595/205651324X16963489202714.

[ref17] Domínguez V. M.; Hernández J. J.; Ramos Á.; Giménez B.; Rodríguez-Fernández J. Exploring the effect of methanol and ethanol on the overall performance and substitution window of a dual-fuel compression-ignition engine fueled with HVO. Fuel 2024, 359, 130529 10.1016/j.fuel.2023.130529.

[ref18] Chintala V.; Subramanian K. A. A comprehensive review on utilization of hydrogen in a compression ignition engine under dual fuel mode. Renewable and Sustainable Energy Reviews 2017, 70, 472–491. 10.1016/j.rser.2016.11.247.

[ref19] Nemmour A.; Inayat A.; Janajreh I.; Ghenai C. Green hydrogen-based E-fuels (E-methane, E-methanol, E-ammonia) to support clean energy transition: A literature review. International Journal of Engine Research 2023, 48, 29011–29033. 10.1016/j.ijhydene.2023.03.240.

[ref20] Ning L.; Duan Q.; Chen Z.; Kou H.; Liu B.; Yang B.; Zeng K. A comparative study on the combustion and emissions of a non-road common rail diesel engine fueled with primary alcohol fuels (methanol, ethanol, and n-butanol)/diesel dual fuel. Fuel 2020, 266, 117034 10.1016/j.fuel.2020.117034.

[ref21] Modiyani R.; Kocher L.; Van Alstine D. G.; Koeberlein E.; Stricker K.; Meckl P.; Shaver G. Effect of intake valve closure modulation on effective compression ratio and gas exchange in turbocharged multi-cylinder engines utilizing EGR. Int. J. Engine Res. 2011, 12, 617–631. 10.1177/1468087411415180.

[ref22] Hountalas D. T.; Zannis T. C.; Mavropoulos G. C. Potential benefits in heavy duty diesel engine performance and emissions from the use of variable compression ratio. SAE Technical Paper 2006.

[ref23] Szybist J. P.; Bunting B. G. The effects of fuel composition and compression ratio on thermal efficiency in an HCCI engine. SAE Transaction 2007, 1398.

[ref24] Stricker K.; Kocher L.; Koeberlein E.; Van Alstine D.; Shaver G. M. Estimation of effective compression ratio for engines utilizing flexible intake valve actuation. Proc. Inst Mech Eng. Part D J. Automob Eng. 2012, 226, 1001–1015. 10.1177/0954407012438024.

[ref25] Xu G.; Jia M.; Li Y.; Chang Y.; Liu H.; Wang T. Evaluation of variable compression ratio (VCR) and variable valve timing (VVT) strategies in a heavy-duty diesel engine with reactivity controlled compression ignition (RCCI) combustion under a wide load range. Fuel 2019, 253, 114–128. 10.1016/j.fuel.2019.05.020.

[ref26] Wang L.; Liang W.; Ma H.; Ji Q.; Sun P.; Liu J. Simulation study on effects of EGR ratio and compression ratio on combustion and emission characteristics of PODE/methanol RCCI engine. Fuel 2023, 334, 126593 10.1016/j.fuel.2022.126593.

[ref27] Saxena M. R.; Maurya R. K. Effect of premixing ratio, injection timing and compression ratio on nanoparticle emissions from dual fuel non-road compression ignition engine fueled with gasoline/methanol (port injection) and diesel (direct injection). Fuel 2017, 203, 894–914. 10.1016/j.fuel.2017.05.015.

[ref28] Aydin H. An innovative research on variable compression ratio in RCCI strategy on a power generator diesel engine using CNG-safflower biodiesel. Energy 2021, 231, 121002 10.1016/j.energy.2021.121002.

[ref29] Sharma P.; Dhar A. Compression ratio influence on combustion and emissions characteristic of hydrogen diesel dual fuel CI engine: Numerical Study. Fuel 2018, 222, 852–858. 10.1016/j.fuel.2018.02.108.

[ref30] Gumber S.; Gurumoorthy A. V. P.; Basile A.; Dalena F.; Chapter 25 - Methanol Economy Versus Hydrogen Economy; Methanol, Elsevier, ISBN 9780444639035) (2018) 661–674.

[ref31] UNECE GTR No. 4. Test procedure for compression-ignition (C.I.) engines and positive ignition (P.I.) engines fuelled with natural gas (NG) or liquefied petroleum gas (LPG) with regard to the emission of pollutants, 2015.

[ref32] Roy M. M.; Tomita E.; Kawahara N.; Harada Y.; Sakane A. An experimental investigation on engine performance and emissions of a supercharged H2-diesel dual-fuel engine. Int. J. Hydrogen Energy 2010, 35, 844–853. 10.1016/j.ijhydene.2009.11.009.

[ref33] ; Rodríguez-Fernández J.; Hernández J. J.; Ramos A.; Barba J.; Domínguez V. M.; Horcajada Ó.; Casero-Alonso V.; Rodríguez-Aragón L. J. Optimization of the Operating Conditions of a Dual CI Engine Fueled with Methanol. SAE Technical Paper 2022–01–0465 (2022).

[ref34] Ghosh A.; Munoz-Munoz N. M.; Chatelain K. P.; Lacoste D. A. Laminar burning velocity of hydrogen, methane, ethane, ethylene, and propane flames at near-cryogenic temperatures. Applications in Energy and Combustion Science 2022, 12, 100094 10.1016/j.jaecs.2022.100094.

[ref35] Sharma P.; Dhar A. Effect of hydrogen fumigation on combustion stability and unregulated emissions in a diesel fuelled compression ignition engine. Applied Energy 2019, 253, 113620 10.1016/j.apenergy.2019.113620.

[ref36] Zhou J. H.; Cheung C. S.; Zhao W. Z.; Leung C. W. Diesel-hydrogen dual-fuel combustion and its impact on unregulated gaseous emissions and particulate emissions under different engine loads and engine speeds. Energy 2016, 94, 110–123. 10.1016/j.energy.2015.10.105.

[ref37] Liew C.; Li H.; Liu S.; Besch M. C.; Ralston B.; Clark N.; Huang Y. Exhaust emissions of a H2-enriched heavy-duty diesel engine equipped with cooled EGR and variable geometry turbocharger. Fuel 2012, 91, 155–163. 10.1016/j.fuel.2011.08.002.

[ref38] Elvers B.; Schütze A. Handbook of Fuels. Energy Sources for Transportation. 2nd ed. Wiley-VCH, 2022.

[ref39] Movileanu C.; Mitu M.; Giurcan V.; Razus D.; Oancea D. Quenching distances, minimum ignition energies and related properties of propane-air-diluent mixtures. Fuel 2020, 274, 117836 10.1016/j.fuel.2020.117836.

[ref40] Lu H.; Yao A.; Yao C.; Chen C.; Wang B. An investigation on the characteristics of and influence factors for NO2 formation in diesel/methanol dual fuel engine. Fuel 2019, 235, 617–626. 10.1016/j.fuel.2018.08.061.

[ref41] Lilik G. K.; Zhang H.; Herreros J. M.; Haworth D. C.; Boehman A. L. Hydrogen assisted diesel combustion. Int. J. Hydrogen Energy 2010, 35, 4382–4398. 10.1016/j.ijhydene.2010.01.105.

[ref42] Zhang Y. Z.; Kung E. H.; Haworth D. C. A PDF method for multidimensional modeling of HCCI engine combustion: effects of turbulence/chemistry interactions on ignition timing and emissions. Proceedings of the Combustion Institute 2005, 30, 2763–2771. 10.1016/j.proci.2004.08.236.

